# Diffusion of Alloying Cobalt Oxide (II, III) into Electrical Steel

**DOI:** 10.3390/ma16186315

**Published:** 2023-09-20

**Authors:** Elmazeg Elgamli, Fatih Anayi

**Affiliations:** Magnetics and Materials Research Group, School of Engineering, Cardiff University, Cardiff CF24 3AA, UK; anayi@cardiff.ac.uk

**Keywords:** electrical steel Si-Fe, cobalt oxide, diffusion technique, (SEM), (EDS), (SST), power losses, permeability

## Abstract

This paper aims to reduce power loss in electrical steel by improving its surface resistivity. The proposed approach involves introducing additional alloying elements through diffusion once the steel sheet reaches the desired thickness. Various effective techniques have been suggested and tested to enhance the resistivity of the strip. The method entails creating a paste by combining powdered diffusing elements with specific solutions, which are then applied to the steel’s surface. After firing the sample, a successful transfer of certain elements to the steel surface is achieved. The amount and distribution of these elements can be controlled by adjusting the paste composition, modifying the firing parameters, and employing subsequent annealing procedures. This study specifically investigates the effectiveness of incorporating cobalt oxide (II, III) into non-oriented silicon iron to mitigate power loss. The experimental samples consist of non-oriented electrical steels with a composition of 2.4 wt% Si-Fe and dimensions of 0.305 mm × 300 mm × 30 mm. Power loss and permeability measurements are conducted using a single strip tester (SST) within a magnetic field range of 0.5 T to 1.7 T. These measurements are performed using an AC magnetic properties measurement system under controlled sinusoidal conditions at various frequencies. The research explores the impact of cobalt oxide (II, III) addition, observing successful diffusion into the steel through the utilization of a paste based on sodium silicate solution. This treatment results in a significant reduction in power loss in the non-oriented material, with power loss reductions of 14% at 400 Hz and 23% at 1 kHz attributed to the elimination of a porous layer containing a high concentration of the diffusing element. The formation of porosity in the cobalt addition was found to be particularly sensitive to firing temperature near the melting point. The diffusion process was examined through scanning electron microscopy (SEM) in combination with energy-dispersive X-ray spectroscopy (EDS). The results demonstrate improved power losses in the coated samples compared with the uncoated ones. In conclusion, this study establishes that the properties of non-oriented electrical steels can be enhanced through a safer process compared with the methods employed by previous researchers.

## 1. Introduction

Electrical steels, also recognized as silicon steels or lamination steels, play an essential role as soft magnetic materials extensively employed in devices like transformers, motors, and generators [1]. These materials effectively confine and amplify magnetic fields, enhancing energy efficiency in these devices [2]. With silicon content potentially reaching 6.5 wt%, electrical steels are typically produced as thin sheets to minimize eddy current loss [3]. While alternative soft magnetic materials, such as amorphous steel, nanomaterials, and soft magnetic composites have emerged in recent years, electrical steels remain a cornerstone in most electrical applications due to their cost-effectiveness, high permeability, commendable energy efficiency, and robust mechanical properties [4].

Electrical steel is a crucial soft magnetic material used in mechanical systems and transformers. It is classified into non-oriented (NO) and grain-oriented (GO) types [5]. Non-oriented (NO) electrical steel exhibits the ability to magnetize and demagnetize easily, rendering it versatile for applications within alternating current (AC) electromagnetic fields, particularly at a frequency of 50 Hz. It serves as the foundation for core production in asynchronous motors, powerful electric rotating machines, limited generators, and other electrical motors involved in energy conversion [6,7,8,9]. Core losses in non-oriented electrical steels primarily comprise hysteresis loss, classical eddy-current loss, and excess loss. The magnetic properties of electrical steel are significantly influenced by factors such as its shape, behavior, microstructure, and texture, particularly in terms of hysteresis loss and excess loss [10]. Eddy current losses, which are caused by electrically conductive core materials, make a substantial contribution to the total core losses [11,12,13]. Developing methods to enhance the core’s resistance to current flow while allowing unrestricted magnetic flux flow is essential [14]. Microstructural factors such as grain size, inclusions, internal stresses, and surface defects have a profound influence on the magnetic properties of electrical steel [15]. Previous research has demonstrated that the magnetic properties of punched, fully processed silicon steels can be enhanced through stress-relief heat treatment [16,17]. In the industry, a widely adopted approach for this treatment involves a conventional annealing method. This technique involves a gradual heating process at a rate of 200 °C per hour, followed by a one-hour hold at a temperature ranging from 700 °C to 800 °C. Afterward, a slow cooling process is applied [17]. The complete conventional treatment procedure, which includes heating, annealing, and cooling, typically has a duration of approximately 12 h. This timeframe allows for the necessary thermal processes to be carried out effectively and ensures that the desired outcomes are achieved in terms of enhancing the magnetic characteristics of the material. Both materials are well suited for high-speed machines with high efficiency, although the CoFe material exhibits superior performance compared with the SiFe material. While the machine parameters and flux pulsations calculated numerically closely align with the measurements, there are notable discrepancies between the calculated and predetermined values for iron losses and friction losses. Nonetheless, meaningful conclusions regarding the comparison of these materials can be drawn based on the measurements [18]. A thorough investigation was conducted to assess the influence of different annealing processes on magnetic laminations composed of CoFe [19]. Tests were performed on stator cores to assess the results. The data gathered from both electromagnetic and micrographic assessments undeniably confirm the importance of utilizing a specialized annealing process to enhance the magnetic and energetic characteristics of the laminations [20]. In this investigation, an extensive comparative analysis of diverse soft magnetic materials, including cobalt–iron alloys, nickel–iron alloys, silicon–iron alloys, amorphous magnetic materials, and SMCs, was carried out. To facilitate rapid and straightforward comparisons, a novel assessment criterion known as the PB2 factor was introduced [21]. Furthermore, this research centered on examining the pivotal role of the annealing procedure and its impact on cobalt–iron and nickel–iron laminations, utilizing magnetic and microscopic assessments. This comprehensive investigation offers valuable insights into the properties and performance of these materials, thereby assisting in the choice and enhancement of soft magnetic materials for diverse applications [22].

### Related Work

The study conducted by [23] yielded significant findings regarding the use of silicon as a diffusant to minimize power loss in NO silicon iron. It was noted that an irregular dispersion of aluminum on the surface, concerning both its concentration and depth of penetration, led to a decrease in the thickness of the layer. Additionally, thin coatings of porosity were detected on both sides of the samples. While this diffusion method had minimal impact on anisotropy constants, it effectively generated the appropriate internal stress [24]. Nonetheless, a significant limitation of this method was the formation of a porous material on the steel side. To regulate the diffusion permeation from the paste into the steel sheet, various diffusions could be restricted, the paste’s structure and depth could be adjusted, or a suitable paste formulation could be employed to ensure a consistent excess of diffusion. By adjusting the firing duration and heat, the amount of soft material could be modified before completely removing the remaining coating. To investigate the influence of a distorted flux waveform on the magnetic properties of an electrical steel sheet, materials with different resistivities throughout their thickness were created by diffusing small quantities of aluminum into electrical steel [25].

Another study demonstrated that manganese could diffuse into steel from a paste containing powdered manganese in a diluted Si oil solution. The addition of Mn resulted in a remarkable reduction of up to 8% in power loss at 1.5 T in NO 2.4 wt% Si Fe. This reduction can be attributed to a straightforward increase in resistivity. These findings highlight the potential of manganese as an effective element for enhancing the magnetic properties of non-oriented silicon iron materials [26].

This research paper presents a novel approach where cobalt oxide (II, III) is utilized as a coating on the surface of silicon iron, followed by diffusion into the laminations through annealing at 890 °C. The diffusion technique and succeeding heat treatment assist in precise control over the concentration of the diffused amount throughout the thickness of the samples. The primary objective of applying the cobalt oxide coating is to enhance the resistivity of the samples, thereby mitigating power losses caused by eddy currents.

The research conducted in this study involves two different phases. In the initial phase, we measured the permeability and power loss of untreated samples of non-oriented (NO) electrical steel, which had been subjected to a treatment involving a cobalt oxide paste. In the subsequent phase, we assessed samples that had been coated with the cobalt oxide paste and compared their permeability and power loss with those of the untreated samples. It is important to clarify that in this context, the term “coating” refers to the application of a paste containing Co_3_O_4_ onto the surface of the silicon–iron strips.

By investigating how the cobalt oxide coating and diffusion process affect the magnetic and energetic properties of the laminations, this research offers valuable insights into the potential advantages of using cobalt oxide as a surface treatment to enhance the performance of silicon–iron materials.

## 2. Materials and Methods

### 2.1. Material

To improve the preparation of cobalt (II, III) oxide (Co_3_O_4_) powder activated, ~99%, with a particle size of <10 μm, and the utilization of sodium silicate solution as an adhesive, particle size analysis has been verified. The particle size of the cobalt (II, III) oxide (Co_3_O_4_) powder will ensure that the particles meet specific requirements.

The material was provided by the supplier (Merck Life Science UK Limited, Gillingham, UK). There are factors such as adhesive strength, compatibility with the cobalt (II, III) oxide (Co_3_O_4_) powder, and long-term stability. Homogeneity improvement required implementing additional mixing or blending techniques to ensure a more homogeneous distribution of the cobalt (II, III) oxide (Co_3_O_4_) powder and sodium silicate solution. These included using mechanical mixers or optimizing the mixing parameters (speed, duration, etc.). The drying process was evaluated by varying parameters such as temperature, duration, and drying method. This helped determine the optimal conditions for achieving the desired adhesion and sufficient drying of the paste. It could enhance the preparation of cobalt (II, III) oxide (Co_3_O_4_) powder and improve the performance and reliability of the resulting paste.

### 2.2. Preparation Samples

#### 2.2.1. Preparation of Uncoated Samples

The non-oriented electrical steel (NOES) M330-35A sample is 30 mm × 0.305 mm × 300 mm (width × thickness × length). Table 1 demonstrates the variation in chemical properties and the essential chemical properties of the M330-35A grade. The steel contains 2.4 wt% silicon (Si) and iron (Fe) as the primary components, along with minimal quantities of other consistent components.

Tata Steel/Cogent Power supplied the M330-35A steel for the study [27]. Additionally, the steel was punched using the original tooling provided by Wingard & Co., located in Baltimore, MA, USA. This information provides transparency about the source and processing of the material and specifies the sources of the data provided in Table 2, indicating that it is derived from manufacturer-provided material data for the M330-35A samples.

**Figure 1:**
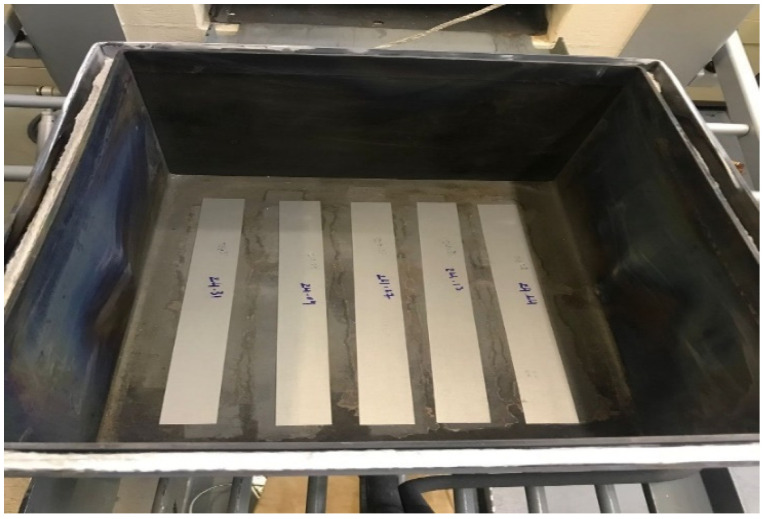
The samples before annealing.

The samples before annealing are shown in Figure 1. Single-strip tester (SST) played a significant role in evaluating the power loss and magnetic permeability of electrical steel. The tests were conducted at various inductions ranging from 0.5 T to 1.7 T and magnetizing frequencies of 50 Hz up to 1 kHz.

#### 2.2.2. Preparation of Samples with Coating

A laboratory-grade glass beaker with a 250 mL capacity played a central role in our mixing process. Employing utmost precision, we accurately measured the powders using an analytical balance, a crucial step to attain precise measurements. For the sodium silicate component, we exercised equal care, employing a calibrated graduated syringe to guarantee the use of accurate volumes.

The subsequent application of the paste onto the steel sheet was facilitated by a carefully chosen nylon-haired brush, characterized by its approximate width. This deliberate selection enabled us to achieve a uniform, smooth application of the paste onto the surface. Throughout the intricate mixing process, we maintained vigilant oversight over the paste’s consistency and viscosity, making necessary adjustments as indicated by our protocols [28]. These adjustments were made possible by regulating the proportions of the powders and liquids, coupled with the judicious use of mixing techniques. By consistently adhering to these specific measurement and application techniques, we conducted the mixing and application of the pastes with unwavering accuracy and uniformity.

##### Samples Coated with Co_3_O_4_

The preparation of samples coated with Co_3_O_4_ involved an equally meticulous approach. We harnessed a modified Co_3_O_4_ powder, mixed in conjunction with sodium silicate solutions provided by “Scientific Laboratory Supplies”. To systematically explore various ratios, the Co_3_O_4_ powder was combined with sodium silicate solutions at concentrations of 0.5 wt% of the paste composition per gram of Co_3_O_4_ powder oxide. The blending of these components was executed with precision using a spiral mixer, a technique that ensured a thorough and consistent coating of the powder with the solution.

To consolidate the integrity of the coatings, a final step involved the careful drying of the coated powder. This was achieved by subjecting the specimens to a controlled environment of 150 °C for a duration of 1 h. This meticulous drying process guaranteed the complete evaporation of any residual liquid, thereby ensuring optimal adherence of the sodium silicate solution to the coated material.

### 2.3. Magnetic Property Measurements by SST

**Figure 2:**
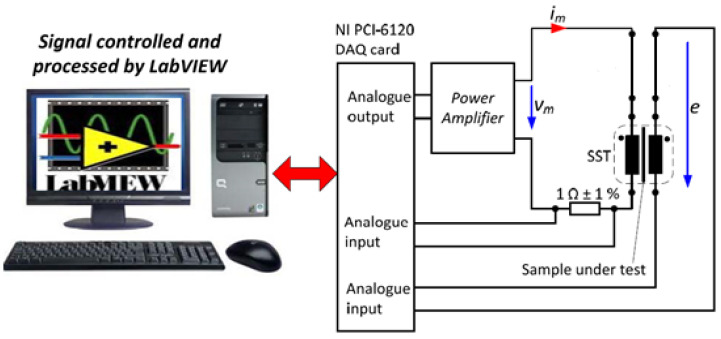
AC magnetic property measurement system [26].

The determination of electrical steel characteristics, such as power loss, is commonly conducted using (SST) or Epstein frames, as recommended by IEC standards (IEC 404-2) [29] and (IEC 404-3) [30]. These measurement systems are specifically designed to assess the permeability and power loss (W/kg) at different magnetizing frequencies. The Magnetics and Materials Research Group at Cardiff University has developed a highly accurate and automated single-strip tester (SST) system, which is widely utilized [31]. The overall system, illustrated in Figure 2, includes a PC with LabVIEW version 2021 preinstalled, a NI PCI-6120 DAQ (data acquisition card), a power amplifier, a 1 ohm shunt resistor (Rshunt), and an air flux-adjusted SST [32,33].

In accordance with the guidelines specified in IEC 404-3, the system utilizes twin vertical yokes constructed from either GOES or NiSi alloy. The primary coil, comprising 865 turns (N1), is wound around the secondary winding, while a secondary coil with 250 turns (N2) is covered around the plastic frame. To facilitate a low reluctance path, a standard Epstein strip-sized sheet measuring 305 mm in length and 30 mm in width is positioned between the yokes. To ensure precise measurement of the desired electrical steel properties, AC tests were conducted under similar conditions, encompassing frequencies reaching from 50 Hz to 1000 Hz. These tests were completed to obtain accurate and reliable information about the characteristics of the electrical steel being evaluated.

### 2.4. Characterisation of Microstructure

Scanning electron microscopy (SEM): SEM is a powerful technique that uses a focused electron beam to scan the sample’s surface. It provides high-resolution images that reveal the topography and morphology of the specimen’s microstructure. SEM was used to examine the distribution of Co diffusion. This technique can help identify how Co has diffused within the material, affecting its microstructure and potentially its properties.

Characterization techniques (SEM) or X-ray spectroscopy (EDS) were used to analyze the morphology, composition, and adhesion properties of the dried cobalt (II, III) oxide (Co_3_O_4_) paste. This would provide a better understanding of the resulting structure and aid in further improvements.

To examine the diffusion effects, the samples were sliced and prepared into square pieces measuring approximately 15–20 mm. These samples were then subjected to examination under (SEM) to determine the distribution of cobalt diffusion.

The investigation focused on the effectiveness of cobalt diffusion on both surfaces of the NOES strips. Concentration profiles were measured using an (EDS) with the assistance of the SEM.

Additionally, SEM was utilized to characterize the composite-coated steel samples. The SEM analysis enabled the observation of inclusions, and (EDS) was employed for elemental investigation of the observed inclusions.

By employing SEM and EDS techniques, the study aimed to gain insights into the diffusion.

The behavior and distribution of cobalt in the NOES samples, as well as the composition of the observed inclusions, were analyzed.

### 2.5. Method for Separating Core Loss

The total loss in electrical steel comprises two main components: hysteresis loss and eddy current loss. The determination of per-cycle hysteresis loss currently involves a distinct method. This method involves measuring the core loss at different frequencies. By extrapolating the magnetization frequency curves to zero frequency for various flux densities, it becomes possible to determine the hysteresis loss per cycle. This per-cycle hysteresis loss represents the energy lost due to hysteresis effects in each cycle [34].

(1) $$\frac{P_{c}}{f}=C_{h}B_{pk}^{n}+C_{e}fB_{pk}{}^{2}$$

The linear equation is used to establish the relationship between the magnetizing frequency (ff) and the maximum flux density (B_pk_). Following this, the core loss data are utilized to plot curves of Pc/f versus f. The three coefficients (Cℎ, C*e*, Ca) were found from the measured core losses at various frequencies and flux density choices. Notably, these curves exhibit a linear relationship and appear as straight lines.

(2) $$\frac{P_{c}}{f}=A+Bf$$

where A = *K*ℎ $B_{pk}^{n}$ means hysteresis loss per cycle and *B* = *Ke* $B_{pk}{}^{2}$.

Further details on determining these coefficients can be found in references [35,36,37].

To calculate the total hysteresis loss, the constant hysteresis power is multiplied by the magnetizing frequency. It is important to note that the hysteresis loss per cycle is independent of frequency. This information is crucial when applying the extrapolation method. However, this assumption holds true only at low frequencies. At higher frequencies, the magnetic area becomes non-unchanging across the lamination, leading to complexities in the calculation.

As a result, within each lamination, the hysteresis loop and hysteresis power losses vary at every point throughout each cycle. Hence, it is more accurate to utilize the extrapolation method for determining core loss separation at low frequencies. Alternatively, when dealing with higher frequencies, the skin effect should be taken into account [38].

### 2.6. Core Loss Separation

The three-term formula employs approaches similar to those used for separating losses into their eddy current and hysteresis components. However, in this specific case, an additional term is introduced to accommodate excess loss.

(3) $$P_{C}=P_{h}+P_{e}+P_{a}\left(W/\mathrm{kg}\right)$$

When Equation (3) is divided by the magnetizing frequency, it yields Equation (4), which provides a representation of the total power losses.

(4) $$\frac{P_{c}}{f}=C_{h}B_{pk}^{n}+C_{e}{\left(fB_{pk}\right)}^{2}+C_{a}{\left(B_{pk}f\right)}^{1.5}$$

In this approach, resembling the two-term separation technique, the first term (Pℎ) represents the hysteresis loss factor, the second term (P*e*) represents the eddy current loss component, and the third term (P*a*) represents the anomalous loss component. The coefficient values for these terms are determined considering factors such as microstructural interactions, magnetic anisotropy, and non-uniformly induced eddy currents [39].

(5) $$\frac{P_{c}}{f}=A+Bf+C\sqrt{f}$$

Another viable approach for determining the coefficients involves plotting the core loss per cycle in opposition to the square root of frequency (√f) instead of frequency (f) for various levels of flux density (B), reaching from the lowest to the maximum frequency [40]. Consequently, Equation (6) can be modified accordingly.

(6) $$\frac{P_{c}}{f}=A+B{\left(\sqrt{f}\right)}^{2}+C\sqrt{f}$$

To determine the coefficients A, B, and C, a nominal curve fitting approach can be employed, where the core loss per frequency (√f) is plotted on the *y*-axis and the square root of frequency (√f) is plotted on the *x*-axis. By comparing Equations (4) and (6), the values of A, B, and C can be found.

(7) $$A=C_{h}B_{pk}^{n}$$

(8) $$B=C_{e}fB_{pk}{}^{2}$$

(9) $$C=C_{a}{\left(fB_{pk}\right)}^{1.5}$$

By employing this method with specific flux densities, the loss factors $C_{h}$, $C_{e},$ *and* $C_{a}$ can be found.

In summary, Equations (1) and (6) highlight the use of the extrapolation technique, which reveals a linear relationship between frequency and the power lost per cycle due to eddy currents. Furthermore, it is understood that the hysteresis loss per cycle remains independent of frequency.

## 3. Results and Discussion

### 3.1. Results of SEM Observations

**Figure 3:**
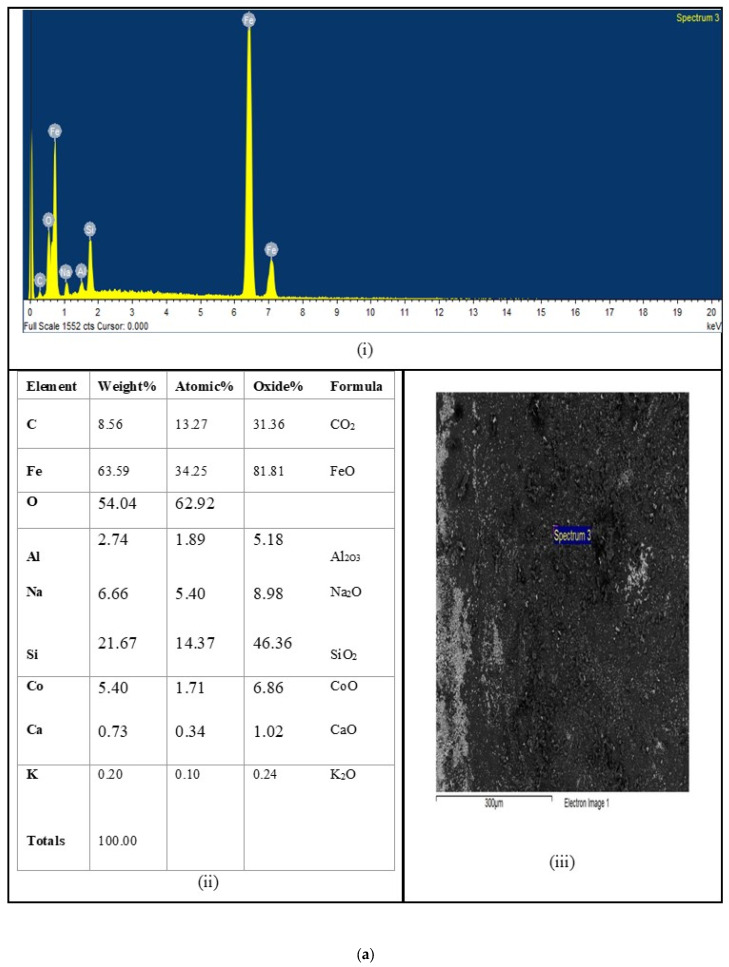

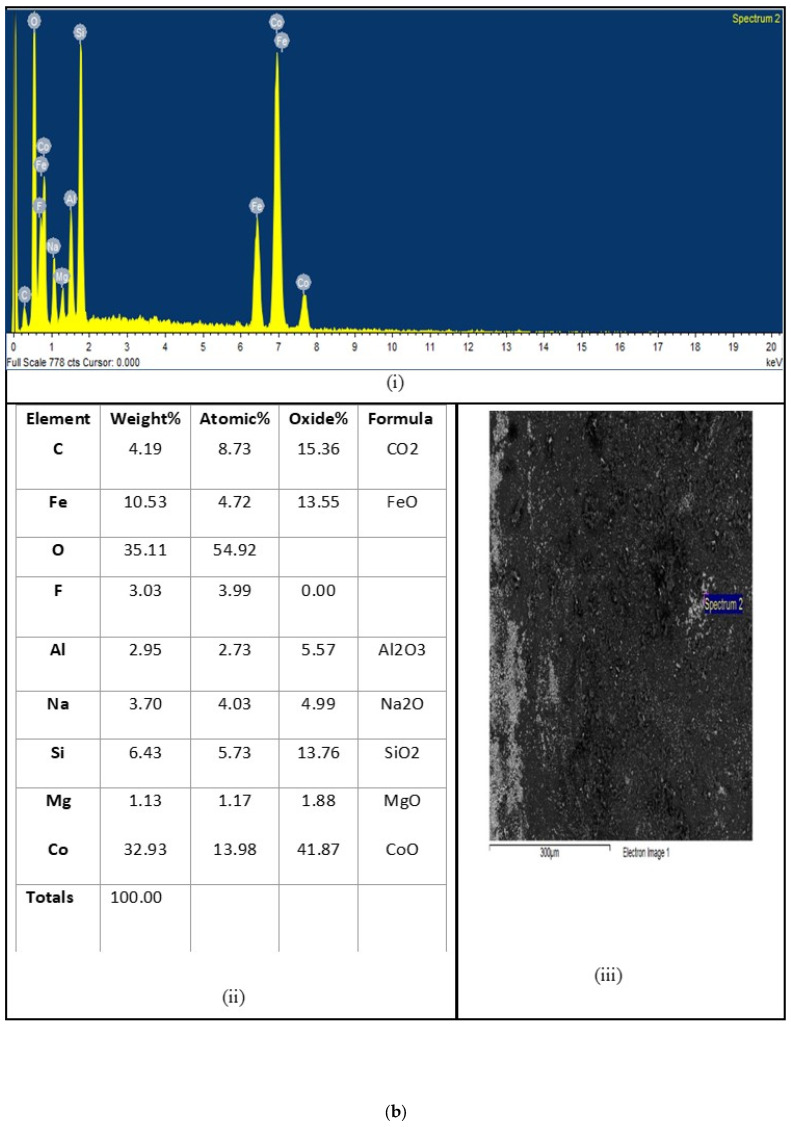

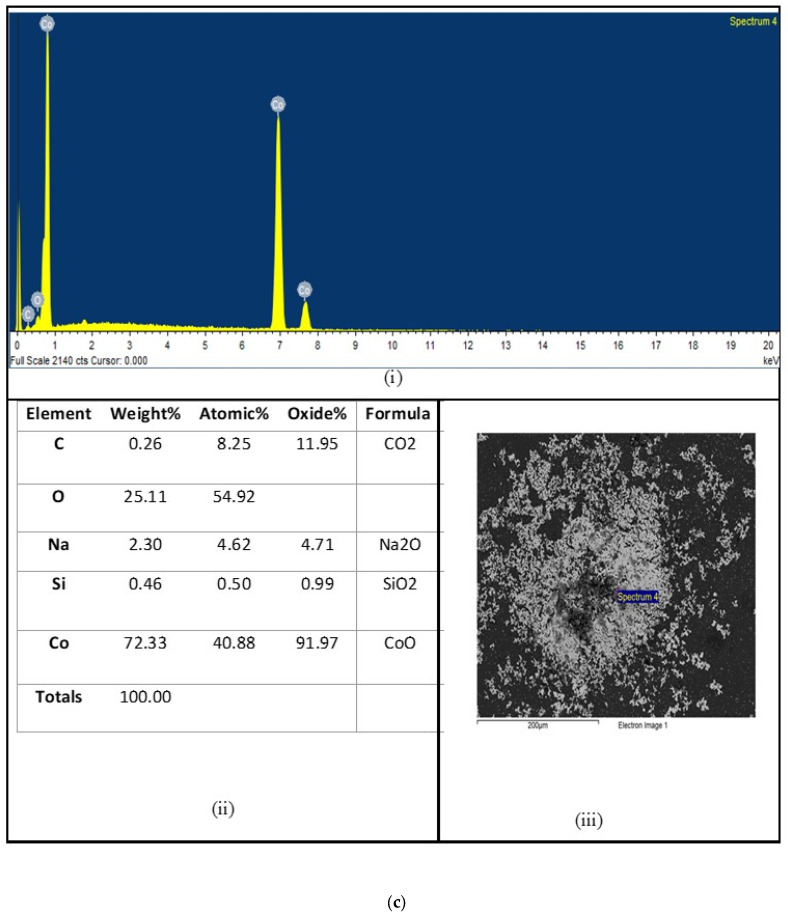
Distribution of elements after heat treatment with EDS analysis of X-rays using SEM. (**i**) EDX spectra of the sample. (**ii**) Table EDS analysis of inclusions from Figure (**iii**). (**iii**) Complex inclusions in the sample of NOES sheet containing w% Co. (**a**) The containing Co 5.40 w%. (**b**) The containing Co 32.93 w%. (**c**) The containing Co 72.33 w%.

Figure 3a–c presents a selection of micrographs that offer a glimpse into the morphology of the samples and the cobalt concentration variation across their thickness. Upon conducting further SEM microstructural examinations, we made a notable discovery: cobalt-rich precipitates were conspicuously absent in the 73 wt% cobalt specimen, a revelation that shed light on the cobalt concentration dynamics throughout their thickness.

These micrographs also feature spectra that enable the determination of cobalt concentration. One of the prominent features of these specimens is the presence of a porous sheet characterized by a high cobalt content near their surfaces. Notably, the limit of cobalt concentration that could be reliably measured often reached the maximum capacity of the rate meter peak. This limitation was typically exceeded in the porous regions where trace levels pushed the measurement to its upper bounds.

The extent of the porous layer proved to be contingent upon the specific composition of the paste used and, to a lesser extent, the firing temperature. Conversely, the composition profile in regions unaffected by porosity exhibited less variation than the thickness of the porous layers but displayed nuanced discrepancies from one spot to another within a given sample.

The porous layer’s maximum width was approximately 72% of the half-sheet thickness, while the minimum width was less than 5%. For context, the paste composition for these two samples comprised 1 mL of sodium silicate solution per gram of cobalt and 1/3 mL per gram, respectively.

It is noteworthy that cobalt concentrations at the porous/nonporous interface exhibited considerable variability within a single sample but typically fell within the range of 5–8%. The penetration depth, measured from the porous/nonporous interface rather than from the steel surface, was approximately 32.9% of the half-sheet thickness when the firing took place at 950 °C. This adjustment is made because it is presumed that the porous layer is relatively magnetically insignificant. These findings illuminate the complex and variable nature of cobalt distribution within the samples, offering valuable insights into the material microstructure and composition dynamics.

### 3.2. Results of Magnetic Testing

All measurements were carried out at 0.5 T to 1.7 T with different frequencies unless otherwise stated. The starting material in each case was 2.4% silicon non-oriented steel. All samples were fired at 890 °C, being kept at the soak temperature for 1 h under an atmosphere of hydrogen. The power loss separation method was applied to calculate the power loss of the non-oriented electrical steel samples. Measurements were conducted using the (SST), and the results are presented in Table 3.

**Figure 4:**
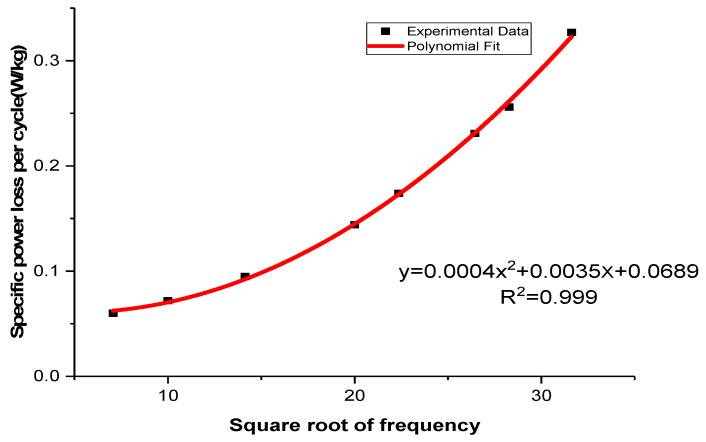
The total power loss per cycle of an uncoated sample made of NO at 1.5 T, with the square root of frequency.

Figure 4 illustrates the relationship between the calculated power loss per cycle and the square root of frequency. To determine the coefficients of the power loss factors, a polynomial curve was fitted to the data using Microsoft Excel 2003.

The residual values of the fitting equation were found to be close to unity, with $R^{2}$ = 0.999, indicating a highly accurate approximation. The results of finding out the power loss components at different frequencies using the equations from Figure 4 are presented in Table 4.

(10) $$A=C_{h}B_{pk}^{n}=0.0689$$

(11) $$B=C_{e}fB_{pk}{}^{2}=0.0004$$

(12) $$C=C_{a}{\left(fB_{pk}\right)}^{1.5}=0.0035$$

**Figure 5:**
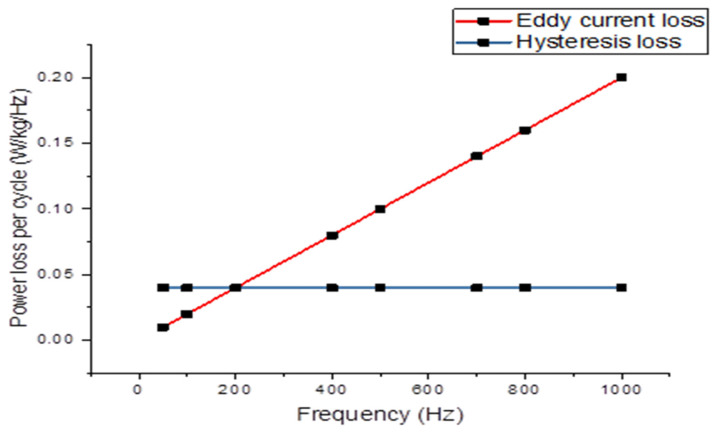
Relationship between eddy current and hysteresis loss per cycle and frequency.

The power loss components, as detailed in Table 4, are calculated based on the coefficients provided in Equation (6). Additionally, Figure 5 depicts the correlation between magnetizing frequency and both hysteresis power loss and eddy current power loss at 1.5 tesla. Hysteresis loss is determined using the static hysteresis loops, while eddy current power loss per cycle is computed using a linear function of the magnetizing frequency.

(13) $$P_{e}=\frac{\pi^{2}}{6\rho }d^{2}f^{2}B_{pk}{}^{2}\;\;\;$$

The power loss of non-oriented steel was measured at various magnetizing frequencies, at 1.5 T. The total power loss per cycle, including the coating, is provided in Table 5.

**Figure 6:**
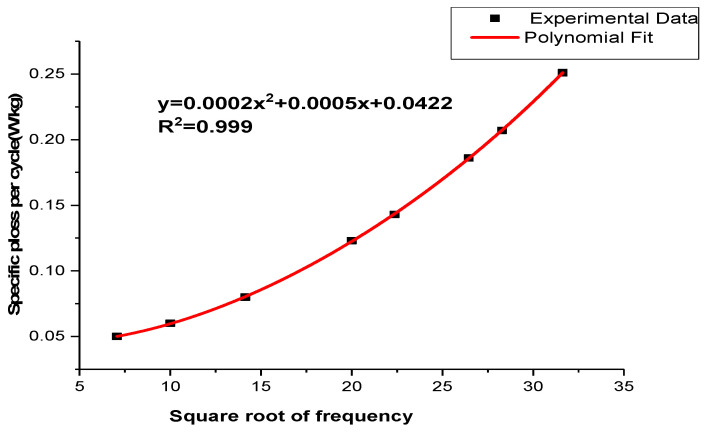
The relationship between the total power loss per cycle of a sample (NO) steel and the square root of frequency at 1.5 T.

Figure 6 displays the relationship between the calculated power loss per cycle and the square root of frequency. By fitting a polynomial curve to this data using Microsoft Excel 2003, we can determine the coefficients of the power loss components with the coating.

Furthermore, Table 6 presents the findings of power loss separation for a magnetic coated sample produced of NOS at 1.5 T, plotted versus the square root of frequency. Evidently, as frequency increases, there is a noticeable escalation in the measured power loss values. This trend is indicative of a significant interplay between frequency and energy dissipation within the materials under investigation. The implications of this trend align with the overarching argument in the study—that cobalt distribution plays a pivotal role in generating a favorable resistivity gradient, leading to the effective reduction of eddy current loss.

Moving forward, the table opens intriguing avenues for exploration. It prompts us to delve into the connection between morphological characteristics or coating thickness and the observed improvement in power loss. The necessity to deepen the quantitative microstructural analysis, in conjunction with the qualitative aspects, is apparent. Moreover, the need to elucidate the mechanisms governing cobalt diffusion is underscored as these intricacies are pivotal to comprehending the observed results.

Table 7 presents a comparison between the eddy current power loss obtained using the extrapolation method (experiment-based) and the eddy current calculated using the conventional formula (theory-based). The results indicate that the calculated eddy current loss in a sample at low induction and low frequency closely aligns with the observed loss obtained through the extrapolation method. The additional loss primarily varies with frequency and field strength while maintaining the same eddy current power loss.

In Table 8, the percentage reduction in power loss for both uncoated and coated samples is shown at different frequencies, confirming that the reduction in power loss occurs at a peak flux density of 1.5 T. The diffusion of cobalt (I, II) oxide leads to an approximate 23% reduction in losses at 1000 Hz and the specified flux density. Additionally, due to significant internal flux distribution effects, even under AC magnetism, the uneven distribution of cobalt causes a resistivity gradient that effectively reduces eddy current loss.

**Figure 7:**
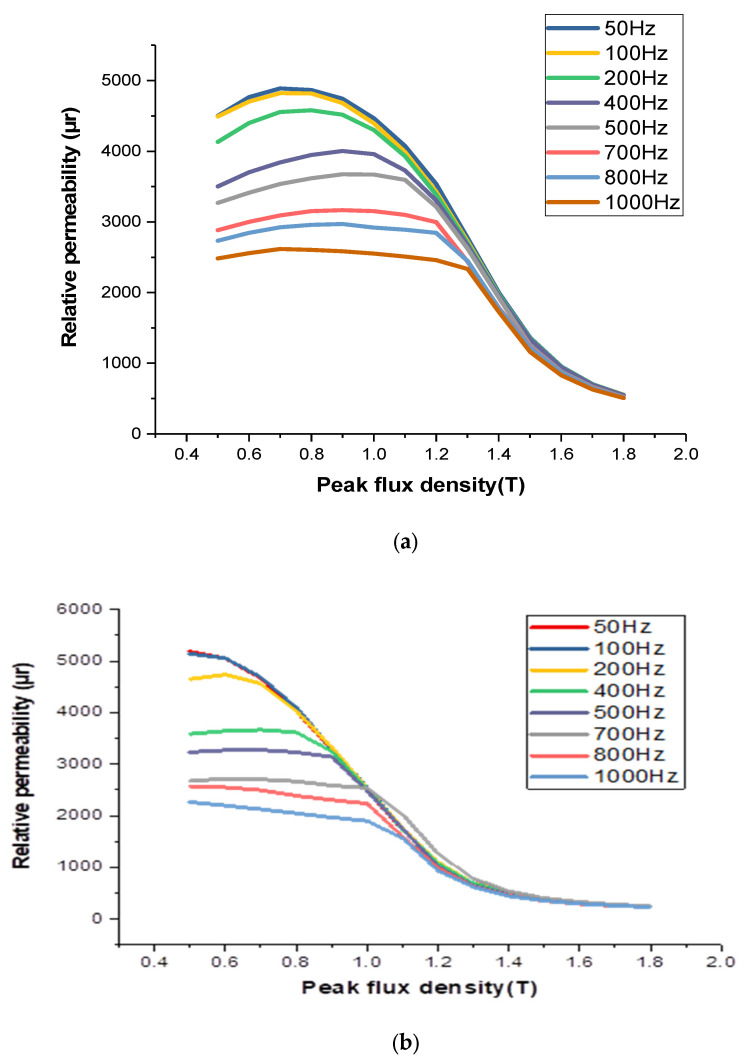
The relative permeability testing for coated and uncoated specimens at different frequencies: (**a**) uncoated sample; (**b**) coated sample.

Figure 7 illustrates the uncoated specimens: a noticeable reduction in permeability is observed as frequencies decrease from 100 Hz to 700 Hz. However, in coated specimens, the opposite trend is observed, with permeability increasing and reaching its highest value at 1 kHz with the coating. Notably, coated samples show the most significant decrease in permeability at 50 Hz with coating and a peak flux density of 0.9 T. The introduction of cobalt (II) oxide through diffusion into the Si-Fe strips leads to an increase in magnetic permeability at the same flux density. This diffusion process also impacts the material’s texture, resulting in a higher anisotropic coefficient, which is reflected in the enhanced relative permeability of the coated material.

### 3.3. Discussion

**Figure 8:**
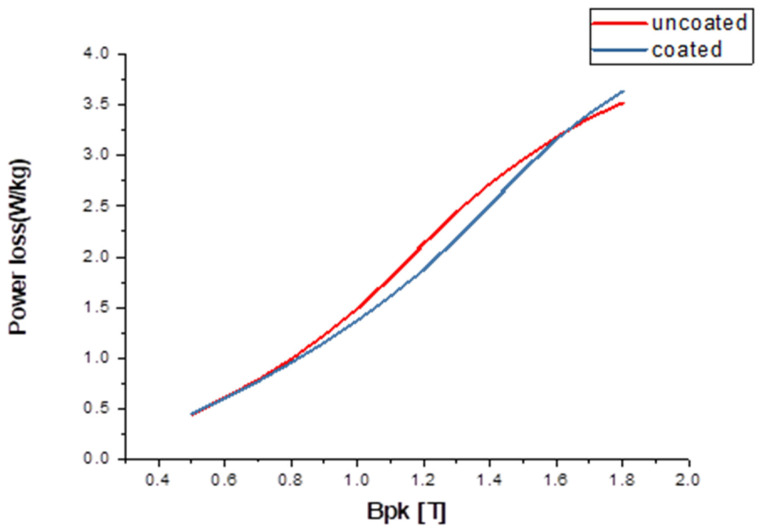
The variation in power loss between the coated and uncoated samples across a range of different flux densities, at a frequency of 50 Hz.

**Figure 9:**
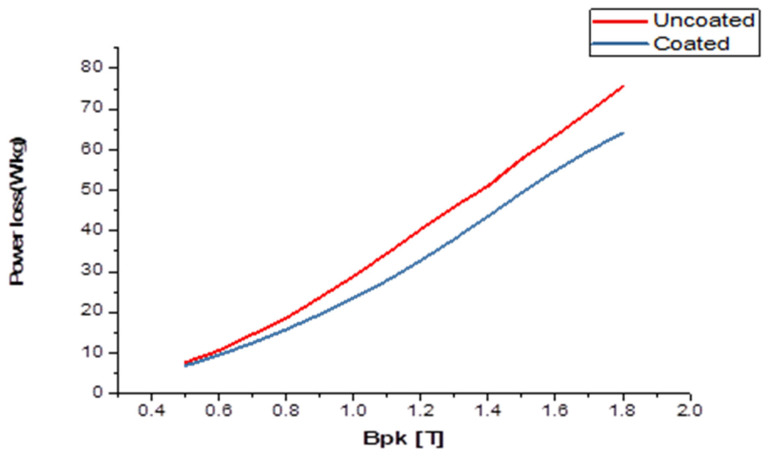
The difference in power loss of the coated and uncoated samples from a magnetic flux density of 0.5 T to 1.7 T at a frequency of 400 Hz.

It was shown that the power loss of the lower grades of NO-SiFe could be reduced under magnetizing frequencies of both 50 Hz and 400 Hz. The value of the reduction was not as great when fired at 890 °C as when fired at higher temperatures during the preliminary experiments. This suggested the incomplete transfer of the cobalt from the paste and the reduction of the penetration depth. Little difference was seen between the samples fired with pastes in the range of 1/3 to 1 mL sodium silicate solution per gram of cobalt. The relationship between the change in power loss at 50 Hz and that at 400 Hz was inconsistent, though in general, the increase in power loss at the higher frequency was not as proportionately large as might have been anticipated. At a frequency of 400 Hz, the coated samples demonstrated a significant improvement in power loss, with the largest improvement reaching 15%. Similarly, at 50 Hz, there was an improvement of 4% in power loss for the coated samples compared with the uncoated ones. Figure 8 depicts the variance in power loss between the coated and uncoated samples at various magnetic flux densities ranging from 0.5 T to 1.7 T at a frequency of 50 Hz: this graph visually presents the effect of the coating on reducing power loss, highlighting the differences observed between the two sample types as the magnetic flux density changes. On the other hand, Figure 9 illustrates the variation in power loss between the coated and uncoated samples at the same range of magnetic flux densities but at a frequency of 400 Hz. These figures provide a visual representation of the power loss improvements achieved through the application of the coating, showcasing the varying effects at changed frequencies and magnetic inductance. The relationship between the behaviors at the two frequencies may be different from that of homogeneous materials because the flux distribution will be different in the two cases. Flux distribution will be more uniform.

The thickness of the sheet at lower frequencies will be concentrated more at the surface, compared with higher frequencies. It is the regions close to the surface of the steel that will be most disturbed by the firing process in terms of concentration changes, stresses set up, and vacancies generated. This might account for the unpredictable behaviors of the samples when magnetized at higher flux densities. Experiments on the higher grade of NOS produced improvements in the power loss of up to 8.6% but with an accompanying increase in the 50 Hz loss.

It is suggested that the paste must contain a considerable amount of porosity. Upon studying the samples using the SEM, it became apparent that the deductions, which had been made from the magnetic testing and observation of the pasting behaviors, were, though basically correct, a considerable oversimplification. Pictures obtained using the scanning electron microscope showed that a considerable amount of porosity was generated in the region close to the sample surfaces. These areas had confused structures but well-defined boundaries within which the cobalt content often exceeded the limit of the analytical scale being used. It is quite likely that intermetallic compounds could be found, but this was not investigated.

**Figure 10:**
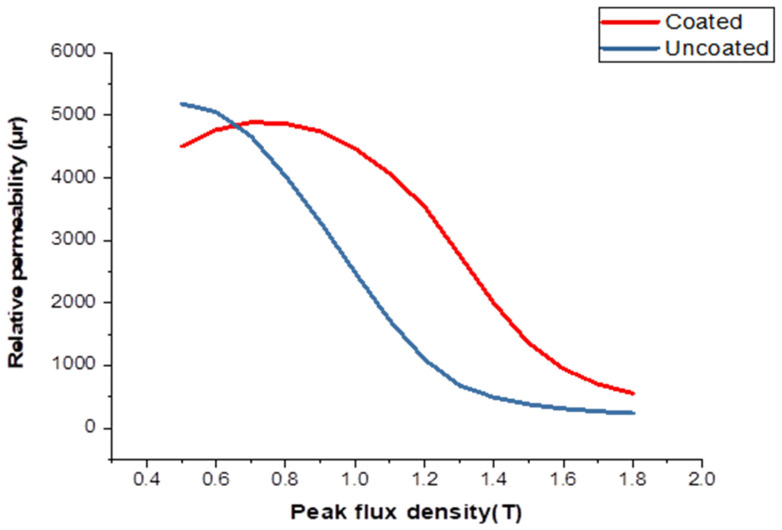
Relative permeability testing of an uncoated and a coated sample at 50 Hz.

**Figure 11:**
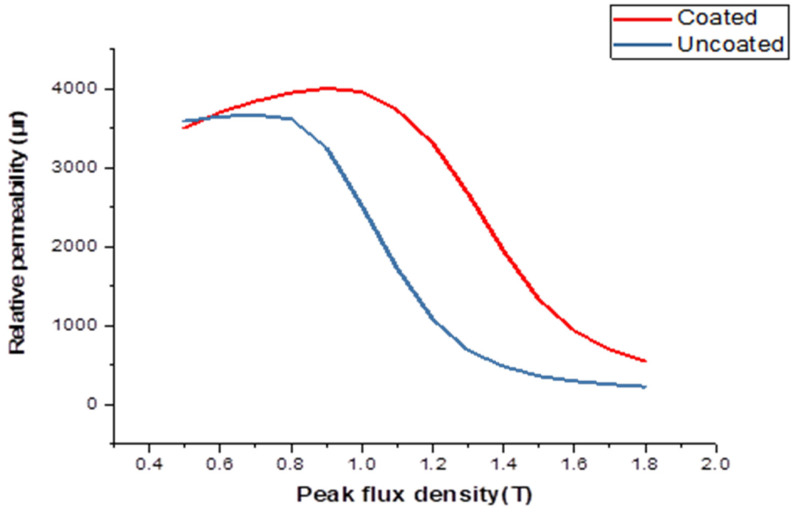
Comparison of relative permeability variation testing of an uncoated and a coated sample at 400 Hz.

Related to the relative rates at which cobalt arrives at the steel surface and diffuses through the metal, this in turn is dependent on the diffusion constants of cobalt in the paste and the metal. While it is unsurprising that diluting the cobalt concentration by increasing the sodium silicate content should lower the activity, the effect of the sodium silicate content is less obvious. The role of reducing the silicate would appear to be a carrier as well as a binder, and for the greatest rate of diffusion through the paste, it must form a coherent matrix. However, as it is not desirable to maximize the rate of diffusion through the paste but rather reduce it to a certain level, it is necessary to evaluate the methods by which this may be achieved. The method of increasing the sodium silicate content of the paste up to a level such that porosity is reduced has the disadvantage of the paste being prone to cracking, resulting in the nonuniform distribution of cobalt as described earlier. Decreasing the silicate content will eventually lead to less coherent coatings. Reducing the silicate content also means that more moisture must be added to the paste so that it will be liquid enough to be brushed on. This will mean that the liquid phase of the paste will be thinner and therefore less able to maintain the powder in suspension, while the paste becomes less tacky. Pastes with less silicate content will become progressively more difficult to brush on evenly with good wetting of the surface. An alternative method is ceramic powder. The viability of this depends on what is to happen to the residual paste after firing because the greater the proportion of sodium silicate and dilutant to cobalt, the greater the amount of residue. If the residue is to be removed, this is unimportant; however, if it is to remain and perhaps be incorporated in an insulating coating, then it is a serious disadvantage. Figure 10 shows relative permeability testing of a sample, uncoated and coated, at 50 Hz. The impact of the porous sheet on the magnetic properties of the sheet would be to effectively reduce the thickness of it by the thickness of the layer as the layer would have very low saturation magnetization and very poor permeability. Comparison of relative permeability variation testing of a sample, uncoated and coated, at 400 Hz is shown in Figure 11.

**Figure 12:**
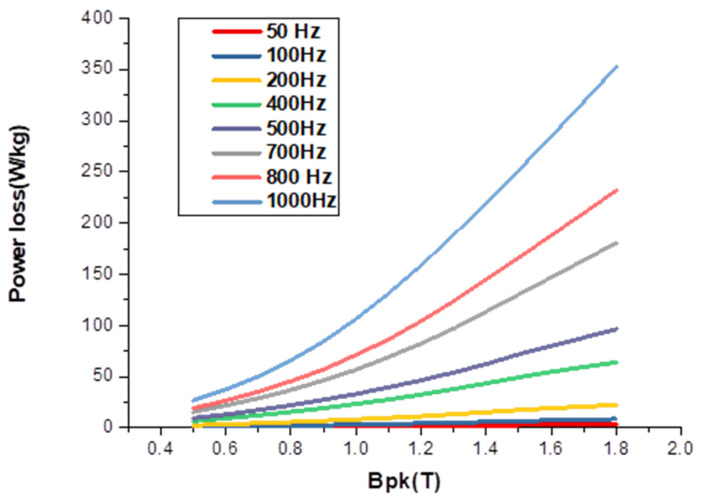
Power loss testing for the coated sample at different frequencies.

The presence of this layer made the removal of the residual layer even more difficult to accomplish as it was often not easy to be certain where the porous layer began and the coating ended. Assuming that the porous layer is practically nonmagnetic, which considering its structure and probable composition would appear to be likely, the magnetic properties of the steel must depend on the thickness of the undamaged layer and the cobalt distribution within it. In the cases in which the porous layer was broad, its thickness varied noticeably from place to place along the strip, indicating either differences in contact resistance between the paste and the steel or inhomogeneity in the paste layer itself. This inconsistency also manifested itself in variations of the concentration gradients in the undamaged part of the sheet. Also, there was less cobalt transferred to the same regions. The factors affecting these profiles become increasingly complex, as the variables already quoted as governing them must be increased to include terms relating to the thickness and diffusion characteristics of the porous layer. It is evident from the SEM photographs, however, that in many cases only a small proportion of the cobalt added to the steel reached the undamaged section of the steel. This would explain why relatively low reductions in the power loss of the materials were recorded as most of the cobalt added was ineffective. As flux density was measured assuming that the thickness of the steel was unchanged from that of the untreated state, the reasons for the apparent increase in magnetization field to achieve the higher values became clear. In the worst cases, the porous layer effectively reduced the thickness of the sheet by up to 30%, as shown in Figure 12.

The generation of the porous layer reduced the thickness of those domains while the roughness of the interfaces provided pinning points for them, thus causing an improvement in the hysteresis loss. In addition, it was displayed that the cobalt cognizing process caused stress within the material, and it is probable that this would have deleterious effects on the magnetic properties. The massive increase in power loss that was reflected after the firing was largely reversed by subsequent annealing, suggesting that it was caused by stress effects. This phenomenon would be far less apparent in the non-oriented material due to it having a far lower stress sensitivity. The impact of further temperature treatment on the fired samples from which the residual coating was removed would be a transfer of some of the cobalt from the cobalt-rich porous regions into the interior of the steel. No mechanism is apparent, however, whereby the porous sheet could be retransformed to a solid phase having the same grain structure as the rest of the steel. The effects of creating resistivity gradients by increasing the cobalt concentration of a sheet near its surfaces are obscured by the production of porous regions and by the stresses which are thereby set up as these. Compressive stress of only a few Mn when applied to this material is more than enough to nullify the benefits obtained from the rise in resistivity brought about by increasing its cobalt content by one or two percent [41].

Compared with the methods used by Ames [42] for siliconization, the benefits of a paste method are that it does not require as much specialized equipment as other processes and it certainly is less hazardous. Although cobalt may be readily transferred from a paste, there are several problems to overcome before it can be usefully exploited—primarily, the production of a stable paste whose diffusive characteristics relative to those of the steel are such that prevent the formation of porous sheets. This could be achieved by controlling the PH values of the sodium silicate solution and its content proportional to that of the cobalt powder. Problems could remain, however, in producing such fired samples from which the residual coating was removed—such as a transfer of some of the cobalt from the cobalt-rich porous regions into the interior of the steel. No mechanism is apparent, however, whereby the porous layer could be reconverted to a solid phase having the same grain structure as the paste and the required adhesive properties together with homogeneity. The other main difficulty with the residual layer is that it must either be made more easily removable after firing or more tenacious so that it can be incorporated into the insulating layer.

## 4. Conclusions

The cobalt paste method has demonstrated its effectiveness in reducing power loss in NO 2.4% SiFe. However, there are challenges associated with the formation of porous layers during the firing process. Overcoming this problem requires formulating a paste with characteristics that prevent the formation of porous layers. Additionally, the paste must exhibit good adhesion during firing to ensure a homogeneous distribution of cobalt over the surface areas. The magnetic properties of samples fired with silicon paste are influenced by the extent of the porous layer, cobalt distribution in nonporous regions, and the stresses within the steel. Power loss relationships between measurements at 50 and 400 Hz can be unpredictable due to variations in flux distributions in nonhomogeneous samples. Residual layers pose another challenge, and they should either be easily removable after firing or thinner, more even, and more tenacious if used as part of the insulating coating. If the latter approach is chosen, strict control over the coating thickness is necessary to limit the amount of silicon entering the steel. Avoiding the formation of porous layers and utilizing annealing to remove any undesirable stresses are crucial steps. If these challenges can be addressed, there is no obvious reason why the method should not be able to produce improvements in oriented materials. This approach would offer significant advantages in terms of convenience and reduced hazards compared with methods relying on cobalt tetrachloride vapor reactions. By investigating the effects of cobalt oxide coating and diffusion methods on the magnetic and active properties of laminations, this research provides valuable insights into the potential benefits of utilizing cobalt oxide as a surface treatment for enhancing the performance of silicon iron materials.

## Figures and Tables

**Table 1:** Chemical composition of M330-35A samples [26].

Grade	C wt.%	Al wt.%	Si wt.%	Fe wt.%
M330	0.0035	0.3000	2.4000	Balance

**Table 2:** Manufacturer-provided material data for M330-35A samples [26].

Grade	Thickness (mm)	Resistivity (μΩcm)	Elastic Modulus, Rolling Directions (N/mm^2^)	Elastic Modulus, Transverse Directions (N/mm^2^)	Yield Strength (N/mm^2^)
M330	0.35	2.4000	200,000	210,000	315

**Table 3:** The power loss of NO steel measured at various magnetizing frequencies, with a total power loss per cycle uncoating at a peak flux density of 1.5 T.

Magnetising Frequency (Hz)	Measured Power Loss (W/kg)	Power Loss per Cycle (W/kg).sec
50	3.00	0.06
100	7.19	0.072
200	19.01	0.096
400	57.80	0.144
500	87.00	0.174
700	163	0.23
1000	327	0.327

**Table 4:** Power loss components of SST of non-oriented steel at different g frequencies at 1.5 T with uncoated sample.

Frequency (Hz)	Measured Power Loss (W/kg)	P_e_ (W/k)	P_h_ (W/kg)	P_a_ (W/kg)	P_c_ = P_e_ + P_h_ + P_a_ (W/kg)	$\mathrm{Error}\;=\;\frac{P\;calculated-P\;measured}{P\;calculated}\times 100\%$
50	3.00	0.1	3.44	0.637	4.17	0.28
100	7.19	0.4	6.89	1.75	9.04	0.20
200	19.01	1.6	13.78	4.95	20.33	0.06
400	57.80	6.4	27.80	14	48.2	−0.12
500	87	10	34.45	39.13	83.58	−0.04
700	163	19.6	48.23	64.82	132.56	−0.22
1000	327	40	68.9	110.77	219.67	−0.48

**Table 5:** The power loss of non-oriented steel at various magnetizing frequencies and a peak flux density of 1.5 T. The measurements include the total power loss per cycle for samples with coating.

Magnetising Frequency (Hz)	Measured Power Loss (W/kg)	Power Loss per Cycle (W/kg).sec
50	2.88	0.058
100	6.78	0.068
200	17.50	0.088
400	49.41	0.123
500	71.99	0.144
700	130.35	0.186
1000	251.80	0.251

**Table 6:** The power loss components of (SST) for non-oriented steel at different frequencies at 1.5 T.

Frequency (Hz)	Measured Power Loss (W/kg)	P_e_ (W/kg)	P_h_ (W/kg)	P_a_ (W/kg)	P_C_ = P_e_ + P_h_ + P_a_ (W/kg)	$\mathrm{Error}\;=\;\frac{P\;calculated-P\;measured}{P\;calculated}\times 100\%$
50	2.88	0.5	2.11	0.177	2.78	−0.03
100	6.78	2	4.22	0.50	6.72	−0.008
200	17.50	8	8.44	1.41	17.85	0.02
400	49.41	32	16.88	4.00	52.88	0.06
500	71.99	50	21.1	5.60	76.70	0.06
700	130.35	98	29.55	9.26	136.80	0.04
1000	251.80	200	42.21	15.81	258.01	0.02

**Table 7:** The results found using the extrapolation method and a comparison with Equation (13).

Magnetising Frequency (Hz)	Eddy Current Power Loss (W/kg) by Extrapolation Method	Eddy Current Power Loss (W/kg) by Equation (13)
50	0.5	0.37
100	2	1.55
200	8	6.88
400	32	24.12
500	50	37.30
700	98	72.25
1000	200	147.45

**Table 8:** Percent reduction in power loss on uncoated and coated specimens at different frequencies at 1.5 T.

Magnetising Frequency (Hz)	Measured Power Loss with Uncoating (W/kg)	Measured Power Loss with Coating. (W/kg)	Reduction in Power Loss (%)
50	3.00	2.88	4
100	7.19	6.78	6
200	19.01	17.50	8
400	57.80	49.41	15
500	87.00	71.99	18
700	163	130.35	21
1000	327	251.80	23

## Data Availability

Not applicable.
